# MicroRNA expression profile during different conditions of hypoxia

**DOI:** 10.18632/oncotarget.26210

**Published:** 2018-10-12

**Authors:** Donato Lacedonia, Giulia Scioscia, Grazia Pia Palladino, Crescenzio Gallo, Giovanna Elisiana Carpagnano, Roberto Sabato, Maria Pia Foschino Barbaro

**Affiliations:** ^1^ Department of Medical and Surgical Sciences, University of Foggia, Policlinico "OO. Riuniti", Foggia, Italy; ^2^ Department of Clinical and Experimental Medicine, University of Foggia, Policlinico "OO. Riuniti", Foggia, Italy

**Keywords:** intermittent hypoxia, chronic hypoxia, pulmonary diseases, biomarkers, microRNA

## Abstract

**Introduction:**

MicroRNAs (miRNAs) are small non coding RNAs which play a role in several cellular processes. MiRNA expression is influenced by oxidative stress, inflammatory cascade and hypoxia. Effects of different types of hypoxia (intermittent and chronic) have been poorly investigated. The aim of this study was to evaluate how intermittent and chronic hypoxia influence the expression of a pool of miRNAs.

**Results:**

Subjects with HI presented higher levels of miR-21, miR-23b, miR-145 and miR-210 compared to the other groups, while higher levels of miR-26 was observed in the HC group. Subjects with HCHI had lower levels of all selected miRNAs. A strong correlation was found between miR-23b and miR-210 and both correlated with PaO2, age and FEV1. MiR-145 is correlated with miR-21 but no correlations were found with other parameters. The level of miR-26a seems to be correlated only with BMI.

**Materials and Methods:**

We used RT-PCR to detect the miRNAs expression in three different models of hypoxemia: intermittent (HI), chronic (HC) and both of them (HCHI). Expression of miRNAs was analyzed using ANOVA and post hoc analysis, moreover, Spearman correlation and Cluster analysis were applied to study the relationship between miRNAs and main clinical parameters.

**Conclusions:**

Intermittent hypoxia induces the expression of some miRNAs more than chronic hypoxia. These miRNAs may play an important role in the development of different diseases usually associated with OSA such as cardiovascular disease. In addition, mechanisms involved in cancer progression may be induced in the presence of chronic and more often intermittent hypoxia.

## INTRODUCTION

MicroRNAs (miRNAs) are 19–22 nucleotide length RNAs that don’t perform protein coding sequences and that mostly regulate the expression of target mRNA at the post-transcriptional level by gene silencing. They play a critical role in several biological mechanisms such as apoptosis, cell differentiation, proliferation and stress response [1]. Thus, changes in miRNA expression are associated with development and progression of human diseases like cancer [2], arterial hypertension [3] and many others systemic diseases. The miRNAs expression can be influenced by different stimuli such as oxidative stress, inflammatory response and hypoxia [4]. Evaluation of changes of miRNAs expression during hypoxia is crucial in understanding the role of miRNAs in many inflammatory diseases and processes. There are two different types of hypoxia: intermittent and continuous. The pathogenic mechanisms underlying these two types of hypoxia in terms of oxidative stress and inflammatory cascade are sometimes completely different, and it was demonstrated by different miRNA expressions according to the type of hypoxia [5]. However, even if many studies were developed to evaluate miRNAs expression during hypoxia, many of them were conducted *in vitro* and little data are now available in human and *in vivo*.

Based on the aforementioned considerations, we hypothesized that specific changes in miRNAs expression would occur in subjects exposed to different types of hypoxia. Therefore, the aim of the present study was to examine the relationship between miRNAs expression and different conditions of hypoxemia.

A variety of hypoxia-regulated miRNAs have been recently identified and they were demonstrated to be a functional link between hypoxia and miRNAs expression, and among them we have chosen a pool of miRNAs which seems to have a strong correlation with hypoxemia: miR-21; miR-23b; miR-26a; miR-145 and miR-210 [6].

To evaluate the expression of miRNAs in different types of hypoxia we have chosen three different diseases representing three different models of hypoxia. Obstructive Sleep Apnea (OSA) is a typical disease that can simulate the condition of intermittent hypoxemia *in vivo*. On the other hand, many diseases can induce chronic hypoxemia, however, the most frequent is the advanced COPD, which is often associated with chronic respiratory failure. Moreover, the association between OSA and COPD is a well recognized disease called Overlap Syndrome (OS), which is characterized by more severe respiratory impairment especially during the night. Thus, these diseases can be an “*in vivo*” model of three different types of hypoxia: intermittent (OSA), continuous (COPD) and continuous-intermittent (OS).

## RESULTS

Table 1 shows demographic and functional characteristics of the study population. Subjects with HC and HCHI were older than HI ones and the control group. BMI was higher in HI and HCHI group than the control and HC, FEV1 and FEV1/FVC was lower in HC and HCHI group compared with HI and control group. Arterial Hypertension and Diabetes mellitus were more frequent comorbidities present in the all subjects.

**Table 1:** General characteristics of study population and miRNAs expression

	Controls		HI		HC		HC+HI		
N (M)	13 (9)		20 (13)		11 (9)		12 (5)		*P*
Former Smoker (%)	*54*		*65*		*54*		*66*		
	*Mean*	*DS*	*Mean*	*DS*	*Mean*	*DS*	*Mean*	*DS*	
Aa (years)	45,71	19,85	55,30	14,43	72,27	8,60	65,67	9,52	<0.01
BMI (Kg/m2)	23,05	1,70	39,23	8,82	27,55	3,91	44,99	9,20	<0.01
pH	7,40	0,02	7,42	0,03	7,42	0,05	7,42	0,02	
paO2 (mmHg)	87,52	8,40	81,22	10,43	55,49	3,05	54,34	4,67	<0.01
paCO2 (mmHg)	38,70	2,90	39,49	3,76	44,11	6,90	46,42	5,07	<0.05
FVC%	105,00	15,43	104,17	21,86	75,40	19,70	82,33	22,04	
FEV1%	106,35	18,56	103,39	26,02	45,50	15,05	78,00	24,23	<0.01
FEV1/FVC	82,60	5,28	80,56	6,37	45,40	9,45	76,11	6,68	<0.01
T90 (%)	0,00	0,00	26,61	25,15	90,20	5,40	62,75	27,00	<0.01
ODI (events/h)	3,10	0,90	46,28	27,80	4,00	0,80	57,95	17,66	<0.01
AHI (evens/h)	2,00	0,70	47,93	24,61	3,50	1,10	60,86	18,51	<0.01
miR-21 (2^-Dct^)	2,05	2,88	19,08	25,73	5,60	6,21	1,77	1,74	0.04
miR-23b (2^-Dct^)	1,77	2,12	7,95	9,29	0,03	0,02	0,96	0,94	<0.01
miR-210 (2^-Dct^)	1,07	1,12	4,27	5,32	0,37	0,34	1,05	1,07	<0.01
miR-26a (2^-Dct^)	1,93	2,96	5,19	5,06	71,61	120,80	0,85	0,47	<0.01
miR-145 (2^-Dct^)	8,83	16,04	31,01	36,76	23,25	27,04	3,72	7,49	0.03

**Figure 1:**
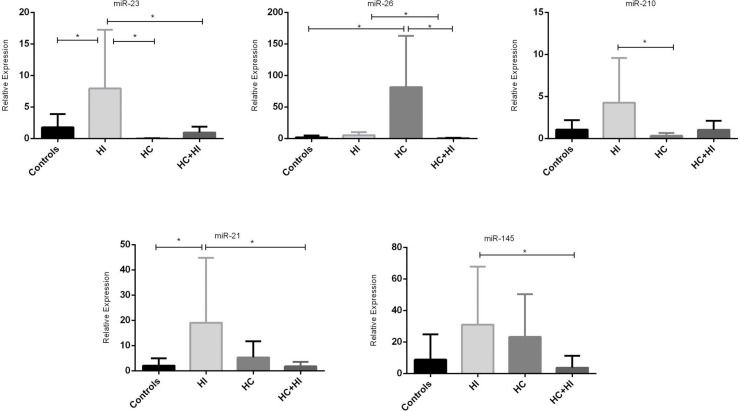
MiRNAs expression in different types of hypoxia (^*^p < 0.05)

Expression of miRNAs was different in the four groups. In particular miR-23 was higher in HI while miR-26 was higher in HC compared to all groups. MiR-210 was higher in HI with respect to HC. MiR-21 was higher in the HI group with respect to controls and the HCHI group. Finally miR-145 was higher in HI patients then in HCHI (Table 1, Figure 1).

**Figure 2:**
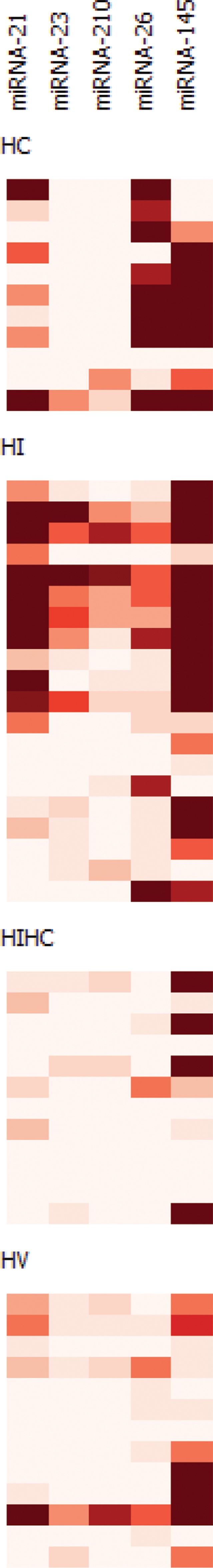
Heat map of miRNAs expression

Usually the expression of all miRNAs was higher in subjects with intermittent hypoxia than in all other groups, but subjects with chronic hypoxia, also showed high expression of miRNAs, while subjects who presented both conditions had lower levels of miRNAs expression, substantially equal to the control group (Figure 2).

**Figure 3:**
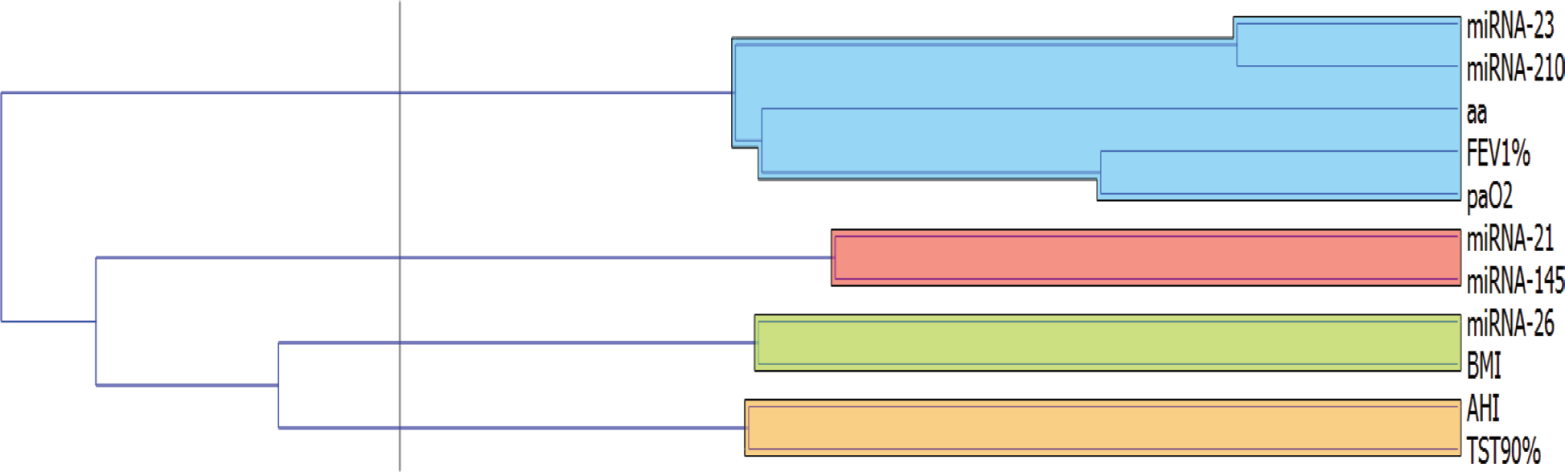
Cluster analysis among miRNAs expression and main clinical parameters

**Figure 4:**
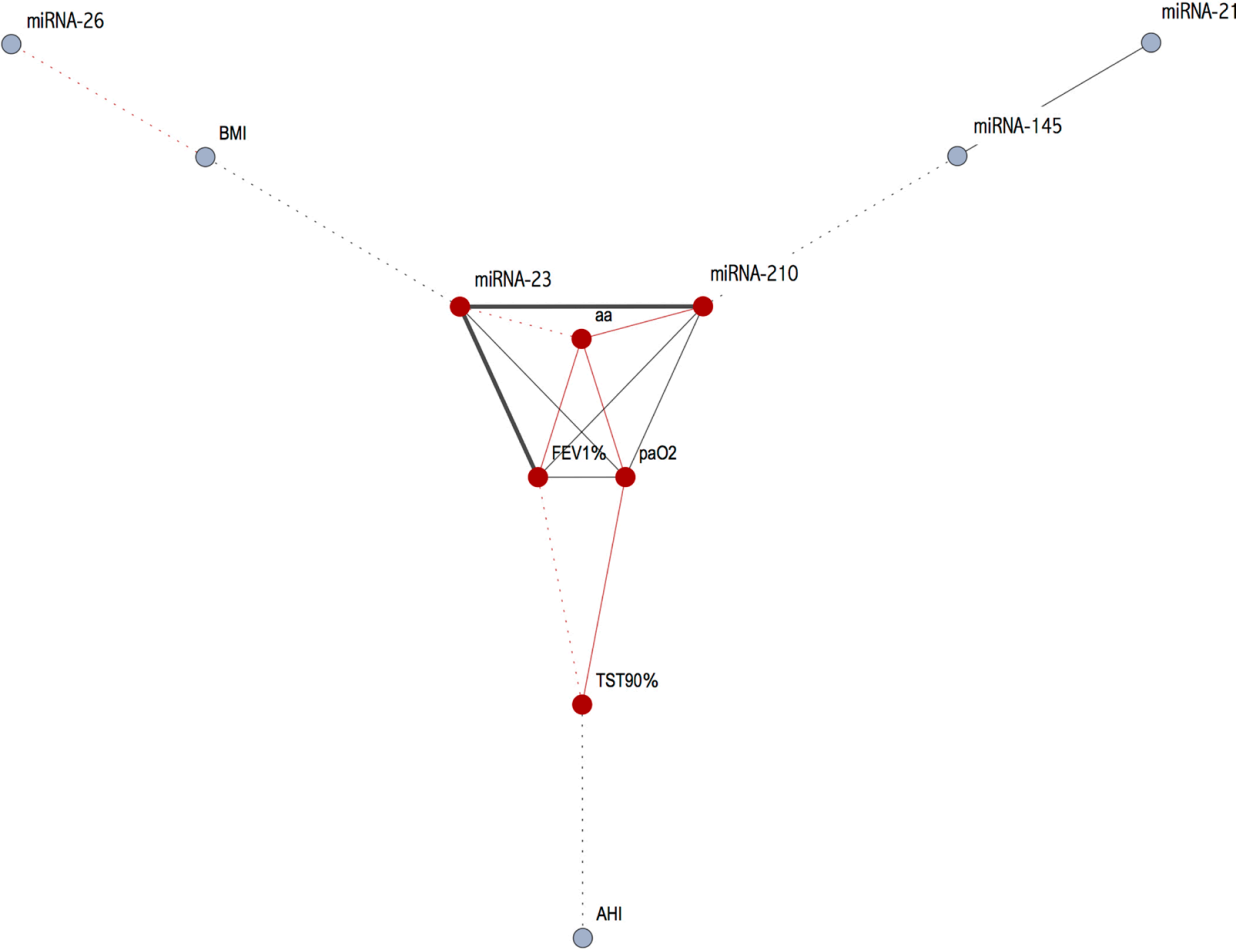
Correlations amongst the miRNAs and the main clinical parameters (black line: positive correlation; red line: negative correlation; dashed line *r* < 0.40; for continuous line the thickness is proportional with correlation index)

Table 2 shows correlations between the different miRNAs and between miRNAs and the main parameters. There was a negative correlation between expression of miR-26, BMI and FEV1 while there were no correlations with other miRNAs. A strong positive correlation was found between miR-23 and miR-210 and both genes were correlated with age, FEV1 and PaO2. Additionally, positive correlation was found between miR-21 and miR-145 and at a lower level with miR-210. These results are better underlined by the cluster analysis (Figures 3 and 4). Curiously, no correlation was found between miRNA expression and main polygraphy parameters such as AHI, ODI and T90.

**Table 2:** Correlation matrix

	miR-21	miR-23b	miR-210	miR-26a	miR-145
*Age*	−0,19	−0,39^*^	−0,48^*^	0,29	0,10
*BMI*	−0,11	0,39^*^	0,22	−0,39^*^	−0,28
*PaO2*	0,14	0,54^*^	0,41^*^	−0,02	0,08
*FEV1%*	0,02	0,71^*^	0,51^*^	−0,27^*^	0,05
*T90%*	−0,02	−0,21	−0,23	0,15	−0,13
*AHI*	0,00	0,25	0,04	−0,15	−0,10
*miR-21*		0,11	0,34^*^	−0,02	0,46^*^
*miR-23a*			0,81^*^	−0,06	0,21
*miR-210*				−0,10	0,37^*^
*miR-26b*					0,05

The value represent Spearman index (^*^p < 0.05).

## DISCUSSION

To our knowledge, this is the first study in which miRNAs expression was investigated in subjects affected by different types of hypoxia. The main finding of this study is that there are two distinct patterns of miR-expression associated with intermittent and chronic hypoxia, and it appears that the first one has a stronger impact on miR-expression than the second, at least amongst miRNAs that we have selected. In particular, four of them (miR-23, miR-210, miR-21 and miR-145) are usually more expressed in the HI group than the other ones analyzed in this study, while only one (miR-26) was highly expressed in subjects with HC. Curiously, when both conditions are present together, there is a reduction of all miRNAs expression, as if there was a sort of “annihilation effect”.

Obstructive sleep apnea (OSA) is a breathing disorder characterized by recurrent obstructions of the upper airway associated with increased inspiratory efforts, intermittent hypoxemia and sleep fragmentation. Extensive research in animal models and humans indicated that these chronic insults contribute to the well characterized mid- and long-term cardiovascular, cognitive and metabolic consequences of OSA.

The mechanisms that regulate gene expression during hypoxia are not fully understood but miRNAs expression seems to have an important role in various processes. MiRNAs are small non-coding RNA molecules which represent approximately 1% to 2% of the eukaryotic transcriptome and have been shown to play critical roles in cell differentiation, proliferation, death, and metabolism and more recently in tumorigenesis. Recent data indicated that hypoxia leaves a specific mark on miRNA profiles in a variety of different cells, with a critical contribution of the hypoxia-inducible factor (HIF). Moreover, at least a subgroup of these hypoxia-regulated miRNAs (HRMs) seems to play a role in cell survival in a low-oxygen environment. It was previously described that expression of some miRNAs (including miR-23b, -24, -26a, -27, -103, -107, -181, -210, and -213) is induced in response to low oxygen. Interestingly, the vast majority of hypoxia-induced miRNAs are also over-expressed in a variety of human tumors. Selective members of this group (such as miR-26a, -107, and -210) decrease pro-apoptotic signaling in a hypoxic environment, suggesting an impact of these transcripts on cancer formation and progression.

During hypoxic conditions, cellular response is modulated by activation of the hypoxia inducible factor (HIF) which regulates the expression of many genes involved in metabolism, angiogenesis, erythropoiesis, cell proliferation, differentiation and apoptosis [7]. Some of these actions are mediated by regulation of miRNA expression so much so that in the last years these hypoxia-inducible miRs were also termed as “hypoxamiRs” [8].

The vast majority of miRNA analyzed in this study (-21, -23b; -26a and -210) are also over-expressed in some types of tumors [9] possibly meaning that hypoxia can induce some of miRNA alterations presented in cancer formation.

MiR-210 is the main miRNA, which is induced under hypoxia [10] and it seems that it is directly regulated by both isoforms of HIF -1α [11, 12] and -2α [13]. MiR-210 is involved in the inhibition of cell proliferation regulating different proteins such as E2F3, member of E2F family transcription factors that have a crucial role in regulation of cell proliferation, differentiation and apoptotic response [14]. However, the target of miR-210 is different in healthy cells with respect to cancer ones in which, on the contrary, miR-210 support cell proliferation [15]. Moreover, it was reported that miR-210 can silence the DNA repair system, so during hypoxic conditions activation of miR-210 may induce genetic instability [16]. Our study shows that during intermittent hypoxia, the level of miR-210 is higher than chronic hypoxia. Recent studies [17] demonstrated that the risk of cancer incidence and mortality increased proportionally with the severity of OSA, thus making it possible to speculate that increase of miRNA level is one of the mechanisms which can sustain tumor progression in patients with OSA.

In this study, we noted that expression of miR-210 is correlated with other two miRNA: -145 and especially with the -23b. The last one is another hypoxia-regulated microRNA involved in apoptosis in different ways and it is up-regulated in some cancers such as pancreas and colon [6]. Up-regulating the expression of miR-23 may help to protect cells from injury-induced apoptotic cell death enabling contribution to cell proliferation [18]. MiRNA-145 has an important role in many inflammatory processes involved in Asthma as well as in COPD [19] but additionally seems to be a promoter of OSA mechanisms such as aortic fibrosis, apoptosis and sympathetic nerve sprouting in a canine model [20]. Our results confirm that intermittent hypoxia, more than chronic hypoxia, increases the level of miR-145 also in human subjects, and this could be a reason why in cardiovascular diseases are more frequent in patients with OSA.

MiRNA-21 and miRNA-26a are both released from endothelial cells [21, 22]. A previous study showed that both miRNA-21 and -26a are expressed after apnea process and speculated that hypoxia induces endothelial cell activation [23, 24]. Our data are in line with these results even if demonstrated that while miR-26a level is higher in chronic hypoxia, miR-21 is more expressed in intermittent hypoxia. This could mean that there is a different endothelial response with respect to type of hypoxemic condition. The increased production of microparticles and miRNA, related to hypoxemia, may be an expression of endothelial dysfunction which is one of the mechanisms underlying the increased cardiovascular risk in OSA patients.

The main limitation of this study is the heterogeneity of population; as often happens *in vivo* studies, some confounding factors such as differences in age, BMI or lung function, may have a critical role in miRNA expression.

In conclusion, we have identified a series of miRNAs involved in different ways in the hypoxic response. This study confirms ‘*in vivo*’ the concept that many studies have already highlighted *in vitro*, that is, the hypoxia induces the expression of different miRNAs, many of which are directly involved in the development of cardiovascular diseases and also in cancer formation and progression. Furthermore, it shows how there is a different response between the condition of intermittent hypoxia compared to the chronic one, and that the first one seems to be, in some cases, much more harmful in terms of stimulating the expression of some miRNA.

## MATERIALS AND METHODS

### Patients

Outpatients referred to clinic for sleep disorders of the Pulmonary Diseases Unit at Ospedali Riuniti of Foggia, were enrolled in the study. Exclusion criteria were: previous diagnosis of OSA, history of cancer, chronic diurnal or nocturnal hypoxemia and already in treatment with oxygen, current smoker, patients with heart failure and recent ischemic heart diseases were also excluded. All patients underwent Spirometry, blood gas analysis and Polysomnography, and accordingly, the results of these tests were classified into three groups:

Intermittent Hypoxia (HI): ODI > 15; diurnal PaO2 ≥ 60 mmHg;

COPD with chronic Hypoxia (HC): ODI < 5; diurnal PaO2 < 60 mmHg;

Intermittent and Chronic Hypoxia (HCHI): ODI > 15; diurnal PaO2 < 60 mmHg;

Subjects who had ODI < 5, normal spirometry and diurnal PaO2 > 60 mmHg were enrolled as Healthy Controls. Thus twenty (20) patients were classified as HI, 11 as HC and 12 with both conditions as HCHI; 13 healthy controls. All the patients had been clinically stable during a period of 6 months prior to testing without exacerbation of COPD or hospital access. The study was approved by the Medical Ethics Committee of Ospedali Riuniti of Foggia and written informed consent was obtained from all study participants.

### Polygraphy

All patients underwent unattended overnight cardio-respiratory monitoring in the sleep laboratory, (Alice PDx; Philips, Amsterdam, the Netherlands). Ronasal flow was measured by a nasal cannula flow, arterial oxygen saturation (SaO2) was measured by a finger probe, abdominal and rib-cage movements were measured by pneumatic sensors. Snoring, sleep position, leg movements, and heart rate were recorded as well. Sleep-disorder breathing was quantified according to standard criteria of the American Academy of Sleep Medicine manual [25]. The examination was considered to be good if there were at least 6 h of registration. A manual score was performed the day after registration by a doctor with experience in sleep disorders.

### Spirometry

Pulmonary function tests were performed in the pulmonary function laboratory using a spirometer (SensorMedics, Yorba Linda, CA, USA).

### Blood gas analysis

An arterial blood sample for the analysis of gases during room-air breathing was drawn with the patient in the sitting position, the day after polygraphy registration and within 1 h of waking up. PaO2, PaCO2 and pH were measured in a blood gas analyser (Model 1312; Instrumentation Laboratory; Milan, Italy). Patients with PaCO2 higher than 45 mmHg were excluded from the study because they could be affected by Obesity Hypoventilations Syndrome (OHS) or Neuromuscolar diseases.

### Collection of blood

Peripheral whole blood was drawn the morning (before the standard breakfast) after the polygraphy. Blood samples were collected in EDTA tubes and centrifuged at 3,000 rpm for 20 min to obtain serum and then stored at −80° C.

### RNA isolation

At the time of the study, commercially available primers were used for 5 mature human miRNAs. The method was optimized for microRNA, and reagents, primers, and probes were obtained from Applied Biosystems. The sequences and nomenclature of the Hypoxia mature microRNAs were obtained from the miRBase Sequence Database version 14.0, released in September 2009. Human RNU-48 was used to normalize all RNA samples. Reverse transcriptase (RT) reactions and real-time PCR (PCR) were performed according to manufacturer protocols. All RT reaction mixtures, including no-template controls and RT-minus controls, were run in duplicate in an Applied Biosystems 7300 real-time PCR instrument (Applied Biosystems, Foster City, CA). Real-time PCR was performed using standard conditions and relative expression was calculated using the comparative cycle threshold method. Total RNA containing small RNA was extracted from serum by using the Trizol reagent in accordance with the manufacturer’s protocol. The concentration and quality of eluted RNA were measured using NanoDrop Spectrophotometer (Thermo Fisher Scientific). RNA purity was evaluated by the ratio of absorbance at OD260/OD280. Reverse transcription and detection of miRNA expression by quantitative Real-time polymerase chain reaction (qRT-PCR) The total RNA was reverse transcribed using a TaqMan MicroRNA RT kit (Thermo Fisher Scientific) according to the manufacturer’s protocol. The identified miRNAs were evaluated in serum by qRT-PCR with Taqman miRNA assay (Thermo Fisher Scientific) according to the manufacturer’s instructions. RNU48 (assay ID 001006) was used as internal control for miRNA quantification in serum to normalize the C_t_ value of miRNA-145 (assay ID 002278), miRNA-21 (assay ID 000397), miRNA-23b (assay ID 000400), miRNA 26a (assay ID 000405) and miRNA-210 (assay ID 000512). Relative expression of targeted miRNAs was computed using the equation 2^−ΔΔCt^, where ΔC_t_ = C_t_ (targeted miRNA) − C_t_ (internal control gene) with respect to the expression of miRNA in the control group. All of the assays were performed in triplicate and one no-template control (NTC) was carried out in each experiment.

### Statistical analysis

Results are presented as mean ± SD. The Kruskal–Wallis test was used (due to data not being normal) to compare differences among groups and post-hoc analysis was employed to evaluate the differences between the expression of miRNA between each group (GraphPad Software, 7825 Fay Avenue, Suite 230, La Jolla, CA 92037 USA).

Hierarchical clustering was performed on miRNA gene profiles’ expression values in order to identify hub genes based on their relationships.

The same technique was employed in order to identify groups of patients by means of a ‘correlation network’, which was built from the reduced data sets connecting only those patients correlating above a given threshold. In this network, edge labels show the correlation ‘intensity’ between nodes, while nodes represent patients. The correlation network obtained shows clusters as ‘communities’, with many edges joining vertices of the same community and comparatively few edges joining vertices of different communities [26, 27]. *P*-value < 0.05 was considered statistically significant.
